# Association of multiple sclerosis with vitiligo: a systematic review and meta-analysis

**DOI:** 10.1038/s41598-020-74298-0

**Published:** 2020-10-20

**Authors:** Meng-Han Shen, Chau Yee Ng, Kuo-Hsuan Chang, Ching-Chi Chi

**Affiliations:** 1 Department of Dermatology, Chang Gung Memorial Hospital, Keelung, Taiwan; 2 College of Medicine, Chang Gung University, Taoyuan, Taiwan; 3 Department of Dermatology, Chang Gung Memorial Hospital, Linkou, Taoyuan, Taiwan; 4 Graduate Institute of Clinical Medical Sciences, Chang Gung University, Taoyuan, Taiwan; 5 Department of Neurology, Chang Gung Memorial Hospital, Linkou, Taoyuan, Taiwan

**Keywords:** Vitiligo, Multiple sclerosis, Epidemiology, Epidemiology

## Abstract

Polyautoimmunity implicates that some autoimmune diseases share common etiopathogenesis. Some studies have reported an association between multiple sclerosis (MS) and vitiligo; meanwhile, other studies have failed to confirm this association. We performed a systemic review and meta-analysis to examine the association of MS with vitiligo. We searched the MEDLINE and Embase databases on March 8, 2020 for relevant case–control, cross-sectional, and cohort studies. The Newcastle–Ottawa Scale was used to evaluate the risk of bias of the included studies. Where applicable, we performed a meta-analysis to calculate the pooled odds ratio (OR) for case–control/cross-sectional studies and risk ratio for cohort studies with 95% confidence interval (CI). Our search identified 285 citations after removing duplicates. Six case–control studies with 12,930 study subjects met our inclusion criteria. Our meta-analysis found no significant association of MS with prevalent vitiligo (pooled OR 1.33; 95% CI 0.80‒2.22). Analysis of the pooled data failed to display any increase of prevalent vitiligo in MS patients compared with controls. Ethnic and genetic factors may play an important role for sporadically observed associations between MS and vitiligo. Future studies of this association should therefore consider stratification by ethnic or genetic factors.

## Introduction

Multiple sclerosis (MS) is an inflammatory, demyelinating disease primarily confined to the central nervous system (CNS)^1^. The clinical characteristics of MS include ophthalmoplegia, trigeminal neuralgia, limb weakness, and sensory and cognitive impairment^2^. The global prevalence varies from 3 to 200 per 100,000, depending on geographic latitude and ethnicity^3^. The etiology of MS remains unclear but may involve B and T cell-mediated inflammation, oxidative stress, cerebrospinal venous insufficiency, and neurodegeneration^4^.

Vitiligo is an acquired depigmenting dermatosis characterized by well-defined chalky white macules or patches. People of all ages and both sexes appear to be equally affected. The prevalence of vitiligo in the general population ranges from 0.06% to 2.28%^5^. The etiology of vitiligo, including its genetic, autoimmune, and biochemical mechanisms, has been studied extensively^6^.

Patients with immune-mediated inflammatory disorders are at increased risk of having more than one type of autoimmune disease^7^. The most common autoimmune comorbidities in MS patients are reportedly psoriasis^8^ and thyroid disease, with a prevalence of 7.7% and 6.4%, respectively^9^. Vitiligo has been associated with diabetes mellitus, thyroid disease, alopecia areata, and pernicious anemia^10^. MS may share similarity with vitiligo regarding immunological, environmental, and genetic factors. However, studies examining the association between MS and vitiligo have reported inconsistent results. We therefore aimed to systemically appraise available data regarding the association of MS with vitiligo.

## Methods

We performed a systematic review and meta-analysis of observational studies on the association between MS and vitiligo, following the Preferred Reporting Items for Systematic Reviews and Meta-Analyses (PRISMA) guidelines^11^. The study protocol was registered with the PROSPERO (CRD42018112959; see https://www.crd.york.ac.uk/PROSPERO/display_record.php?RecordID=112959).

### Inclusion and exclusion criteria

We included case–control, cross-sectional, and cohort studies quantifying the association of MS with vitiligo, and only studies that included a case group of patients with MS and a control group of individuals without MS. The study outcome was the odds ratio (OR) and risk ratio (RR) of vitiligo in association to MS for case–control/cross-sectional and cohort studies, respectively. We excluded studies that did not include a control group or lacked sufficient data relevant to the calculation of OR or RR.

### Literature search and study selection

A literature search of the MEDLINE and Embase databases was performed on March 8, 2020. The search strategy is listed in Table 1. Two authors (MS and CN) independently screened the titles and abstracts of the articles retrieved by the search and obtained the full text of potentially eligible studies to evaluate if they met the inclusion criteria. If the two authors had different judgement, such discrepancies were resolved by consulting a third author (CC).

**Table 1:** Search strategy.

Database	Search strategy
MEDLINE	#1. exp Multiple Sclerosis/
	#2. multiple sclerosis.mp
	#3. disseminated sclerosis.mp
	#4. or/#1-#3
	#5. exp Vitiligo/
	#6. vitiligo.mp
	#7. leucoderma.mp
	#8. leukoderma.mp
	#9. or/#5-#8
	#10. #4 and #9
Embase	#1. ‘multiple sclerosis'/exp OR 'multiple sclerosis'
	#2. ‘disseminated sclerosis'
	#3. #1 OR #2
	#4. ‘vitiligo'/exp OR ‘vitiligo'
	#5. ‘leucoderma'
	#6. ‘leukoderma'
	#7. #4 OR #5 OR #6
	#8. #3 AND #7

### Data extraction

For each included study, we extracted data including first author, publication year, country, study design, confounders that were matched or adjusted in the statistical analysis, number of patients in the case and control groups, the definition of cases and controls, the selection of controls, and quantitative estimates on the association of MS with vitiligo.

### Assessment of risk of bias

The risk of bias of included studies was assessed using the Newcastle–Ottawa Scale (NOS)^12^. Two authors (MS and CC) interpreted the NOS results of each included article. Three domains were evaluated for cross-sectional and case–control studies, including selection of the studies (adequate definition and representativeness of the cases, selection and sufficient definition of the controls), comparability, and exposure (ascertainment, the same method for ascertainment of cases and controls, non-response rate). Similarly, three domains were evaluated for the cohort studies, including selection of the studies, comparability, and outcome. Each domain could be rated as ‘low risk’, ‘uncertain risk’, or ‘high risk’. For example, if a study controlled for confounding factors such as age and sex by either matching or statistical adjustment, we considered it at low risk of bias for comparability.

### Statistical analysis

The Review Manager 5.3 software was used to perform the meta-analysis^13^. For case–control/cross-sectional studies, we calculated the pooled OR of vitiligo in MS patients. Heterogeneity was assessed by using the *I*^2^ statistic. If the *I*^2^ value is more than 50%, it represents moderate heterogeneity^14^. If *I*^2^ was lower than 50%, we chose a fixed-effect model meta-analysis. If *I*^2^ was higher than 50%, we performed a random-effects model meta-analysis^15^. Potential publication bias was assessed by examining funnel plots when at least 10 studies had been included^16,17^. All statistical tests were two-sided, and a probability (*P*) value < 0.05 was considered statistically significant.

## Results

### Characteristics of included studies

**Figure 1:**
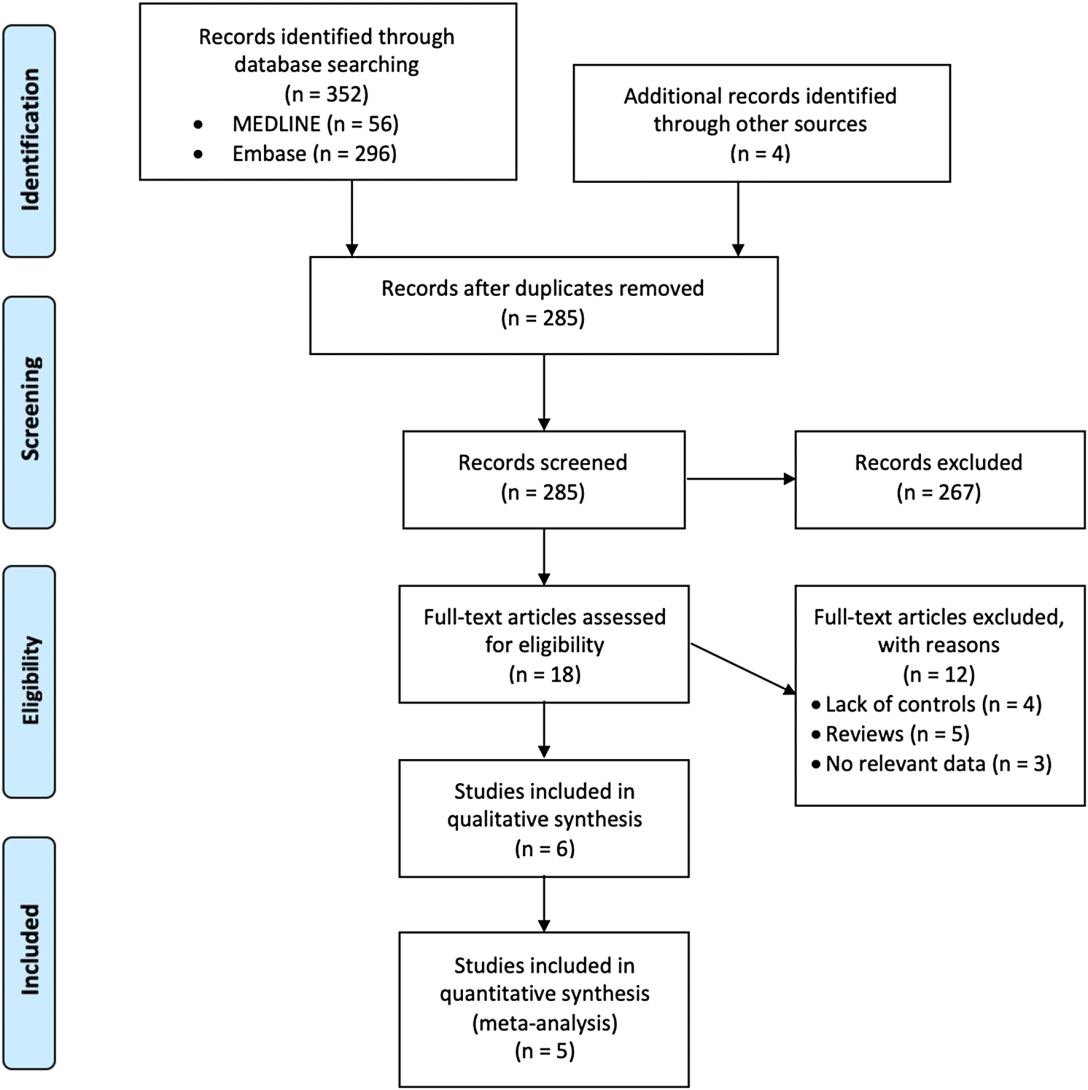
PRISMA study flow diagram.

After removing duplicates, our systematic search identified 285 studies. Ultimately, a total of six case–control studies with 12,930 study subjects that investigated the association of MS with vitiligo met our inclusion criteria^18–23^. We found no relevant cross-sectional or cohort studies. The selection process and reasons for exclusion are illustrated in Fig. 1. The characteristics of the included studies are summarized in Table 2. These studies were all case–control studies performed in Western countries. The sample size ranged from 101 to 5,296, and the mean age of study subjects varied between 39.0 and 55.2 years.

**Table 2:** Characteristic of included studies.

First author, year, country	Study design	Diagnostic criteria: MS identified by	Outcome definition: vitiligo identified by	Cases (% female and mean age)	Controls (% female and mean age)	Results
						Crude OR (95% CI)
Seyfert, 1990, Germany^19^	Case–control study (matched by age)	Poser criteria	Medical chart review	101	97	(0/101)/(0/97)
Handerson, 2000, Australia^20^	Case–control (Medical chart review/ Standardized administered questionnaires)	ICD selection code 340, 205 from Royal Brisbane Hospital Department of Neurology, database from 1992 to 1997, diagnosis of clinical definite or laboratory-support	Mailed questionnaire Controls were selected from persons living in the same street as the cases	117 (71%, 46.2 years)	221 (60%, 51.0 years)	(0/117)/(2/219)
Laroni, 2006, Italy^21^	Case–control (1:1 ratio, matched by gender, and age)	Diagnosis by Poser criteria and the patient was followed up by neurologist	Medical chart review	245 (68%, 39 ± 11 years)	245 (61%, 37 ± 12 years)	0.33 (0.03, 3.20)
Ramagopalan, 2007, Canada^22^	Case–control (Medical chart review/Standardized administered questionnaires)	Poser and/or McDonald 2005 criteria	Medical chart review/Standardized administered questionnaires	5031 (72%, 55.2 years)	2707 spousal controls (MS simplex family: 23%, 59 years; MS multiplex family: 33%, 56.5 years)	1.57 (0.82–3.04)
Langer-Gould, 2010, United States/ Northern California^23^	Case–control (1:5 ratio, matched by gender, age and Kaiser membership characteristic)	Diagnostic codes entered by neurologists and primary care physicians, MS- specific medications, MRI and or lumbar puncture (codes/criteria not specified)	At least two diagnostic codes entered by a specialist/ KPNC (codes not specified)	5296 (75%, 54.5 years)	26,478 (75%, 54.5 years)	0.86 (0.33–2.23)
Deretzi, 2015, Greek^18^	Case–control (matched by gender and age)	McDonald 2005 criteria	Medical chart review, Standardized questionnaires by three neurologists in a structured face-to-face interview	2140 (68%, 33.7 ± 10.2 years)	1580 (65%, not reported)	10.40 (1.37, 79.15)

*CI* confidence interval, *MS* multiple sclerosis, *OR* odds ratio.

**Figure 2:**
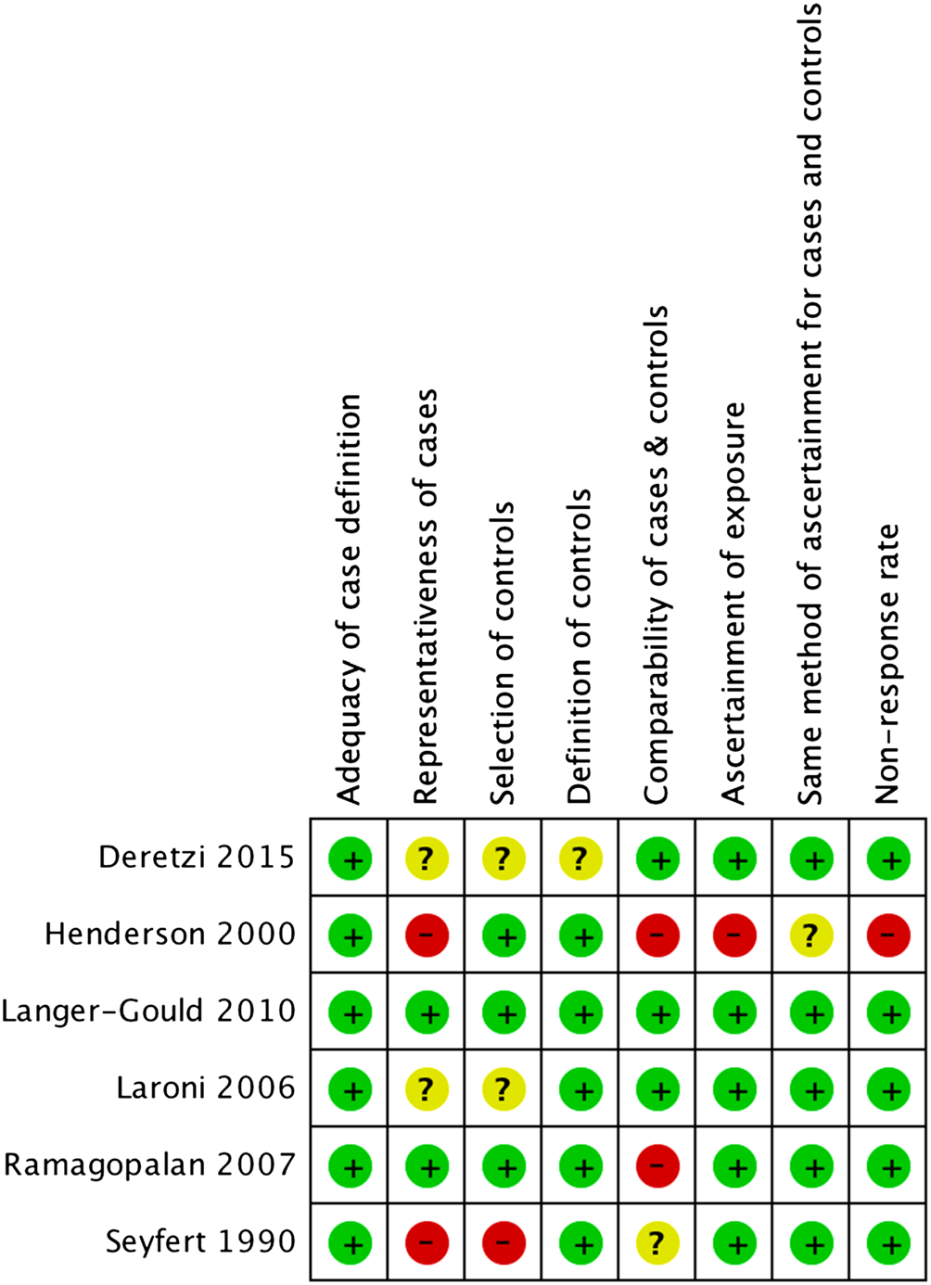
Risk of bias of included case–control studies.

The risk of bias evaluation for included case–control studies is summarized in Fig. 2. All studies had utilized the diagnostic criteria for MS, such as Poser criteria and McDonald criteria of 2005^24,25^. Two studies were rated as having a high risk of bias in the representativeness of cases because the study individuals were from a single hospital^19,20^. One study was rated as having a high risk of bias as to the selection of controls because of recruiting only from a single hospital^19^. Two studies were rated as having a high risk of bias regarding the comparability of cases and controls due to the lack of control for age or gender^20,22^. The study by Handerson et al. was rated as having a high risk of bias with regard to the method of ascertainment of cases and controls, which had been based on written self-reporting, and also with regard to non-response rate, since the non-response rates differed markedly between the two groups (82% in cases, 68% in the control group)^20^.

### Odds for prevalent vitiligo in patients with multiple sclerosis in case–control studies

**Figure 3:**
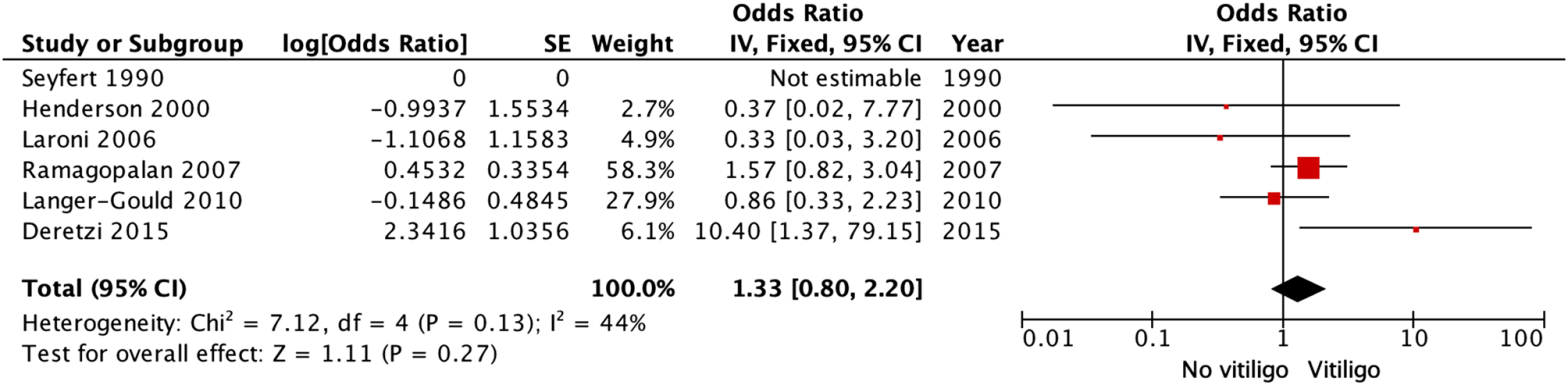
Odds for prevalent vitiligo in multiple sclerosis patients.

**Figure 4:**
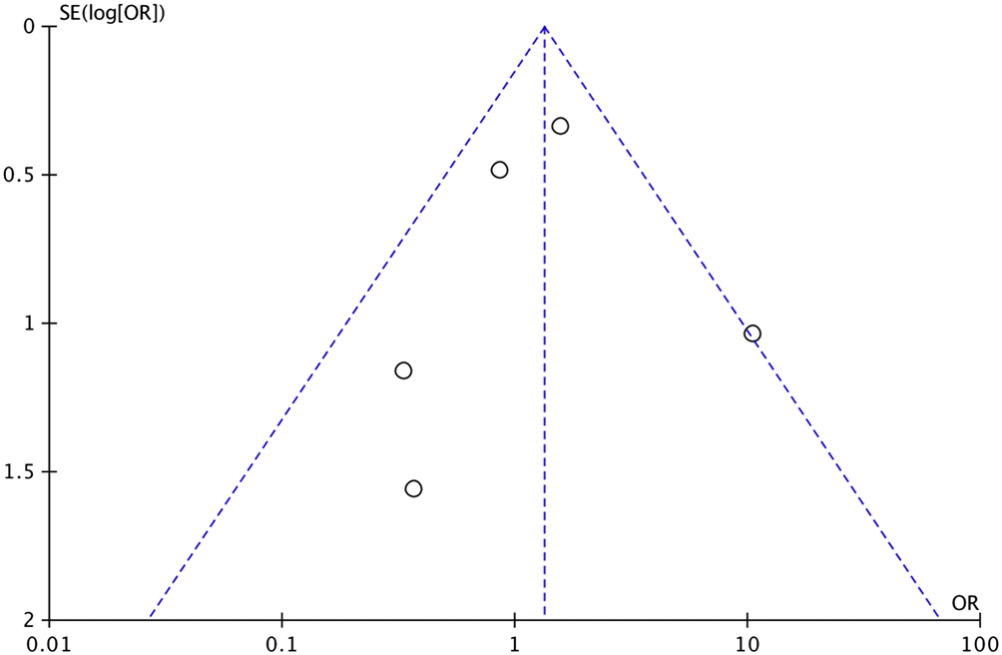
Funnel plot of multiple sclerosis and prevalence of vitiligo. The symmetric distribution suggested no publication bias. However, the number of included studies was less than 10.

In the Seyfert 1990 study, there were no cases of MS with vitiligo^19^; therefore, we could not calculate the OR. Only one study^18^ reported a positive association of MS with prevalent vitiligo. As shown in Fig. 3, our meta-analysis was based on five case–control studies, involving a total of 12,829 MS patients and 31,231 controls^18,20–23^. No significant association of MS with prevalent vitiligo was found (pooled OR 1.33; 95% confidence interval [CI] 0.80‒2.22; *I*^2^ = 44%). No publication bias was detected based on the symmetry of the funnel plot (Fig. 4).

## Discussion

Polyautoimmunity in patients with MS has been proposed. Different types of autoimmune diseases may have shared pathways and may therefore be associated with each other^26^. By using a Greek population, Deretzi and colleagues found a significantly higher prevalence of vitiligo in MS patients than in controls^18^. However, the present meta-analysis did not support an association between MS and vitiligo. As discussed below, the discrepancy in observations pertaining to the association between MS and vitiligo might reflect various factors such as immunologic, environmental, and genetic factors.

The pathophysiology of MS includes peripheral and CNS-compartmentalized inflammatory mechanisms. CD4 + T cells and CD8 + T cells cause neuro-axonal injury both peripherally and in the CNS. Dysregulation of the blood–brain barrier by pro-inflammatory cytokines, interleukin (IL)-17, and chemokines promotes neurodegeneration. Dysfunction of regulatory T cells (Tregs) and an abnormal B cell cytokine response can lead to an aberrant T-cell response^27^. Genome-wide studies show that IL-2 receptor α (IL2RA) gene and IL-7 receptor α gene play a role in the pathogenesis of MS^28^.

The pathogenesis of vitiligo includes genetic, autoimmune, and biochemical mechanisms. Increasing evidence in recent years suggests that vitiligo is caused by cytotoxic response of CD8 + T cells that induce apoptosis of melanocytes^29^. Gene expression profiling in lesional skin of patients and a mouse model of vitiligo indicates an increase expression of interferon gamma and interferon gamma induced genes^30^. Oxidative stress may also contribute to melanocyte destruction^31^. Studies also suggest that lesional skin has increased levels of interferon-gamma, IL-10, and IL-17^6^. Genome-wide studies show susceptible genes associated with vitiligo including NLRP1, XBP1, and IL2RA genes^32^. From a pathophysiology perspective, although the major target cells and antigens are different, MS and vitiligo may have a shared pathologic pathway including increased levels of interferon gamma and IL-17, oxidative stress, and IL2RA gene.

Potential environmental factors associated with MS include smoking^33^, vitamin D deficiency^34^, and decreased exposure to sunlight^35^. Vitamin D plays a role in immune regulation and gene expression. It has a role in the induction of B lymphocyte apoptosis and pro-inflammatory cytokine suppression^36^. Sunlight is the main source of vitamin D, and an inverse association among exposure to sunlight and the incidence of MS has been identified^35^. Vitamin D also plays an important role in the development of secondary autoimmune illnesses in patients with vitiligo^37^.

MS is regulated by multi-genetic and epigenetic factors^38,39^. Human leukocyte antigen (HLA) studies and genome-wide association studies (GWAS) from different ethnic groups have identified several genes associated with MS^40,41^. A study using a similar methodology has been performed on vitiligo patients^42^. Disease-associated genetic loci shared between MS and vitiligo have been identified^40,43^. Individuals who carry the HLA-DRB1 gene may have an increased risk of developing both MS and vitiligo^44,45^. In Greece, the HLA-DRB1 gene has been observed to be significantly more common in MS patients than healthy controls^46^. However, after reviewing studies with data on MS and vitiligo in Greece, current evidence is insufficient to explain why MS patients in Greece have a higher odds for vitiligo compared with other ethnic groups. The diversity of different genetic and epigenetic factors in different ethnicities may, at least in part, explain the inconsistent results reported regarding the association between MS and vitiligo.

Treatments of MS include that for acute relapses and long-term disease modifying therapies. Steroid pulse therapy for 3 to 5 days is applied to control the acute relapses of MS. Disease modifying therapies including injection of interferon beta, glatiramer acetate, natalizumab, alemtuzumab, ocrelizumab, and oral medications such as fingolimod, teriflunomide, dimethyl fumarate, and cladribine can reduce the annualized relapse rate and slow disability progression^47^. Interferon beta was mainly used to treat MS from 1993 to 2010 before the first oral medication fingolimod came out. Alemtuzumab was launched into the market in November 2014. Despite reports on onset of vitiligo following treatments with interferon beta-1a and alemtuzumab, no evidence indicated that prevalent vitiligo in MS patients was affected during these time periods^48,49^.

This systematic review has limitations. First, as all the included studies were from Western countries, the results of this review may not be extrapolated to other ethnic groups. Second, in our study, the enrolled studies did not report what kinds of treatment the patients received, and it could be an important source of bias that may affect the association between MS and vitiligo. Therefore, we advocate studies on different ethnic groups involving MS probands and their family members, and studies that consider treatments of MS. Third, we identified no relevant cohort studies and thus the risk for incident vitiligo among patients with MS remains unclear.

In conclusion, the current evidence does not support an association of MS with prevalent vitiligo. To further explore the association, future national studies could provide more information about genetic and environmental influence on the association between MS and vitiligo. GWAS studies of vitiligo or MS in Greece may provide the clue to explain the association between MS and vitiligo. It is still worth being aware of the possibility of vitiligo in MS patients.
